# The Role of Intentional Strength in Shaping the Sense of Agency

**DOI:** 10.3389/fpsyg.2019.01124

**Published:** 2019-05-21

**Authors:** Samantha Antusch, Henk Aarts, Ruud Custers

**Affiliations:** ^1^Department of Psychology, Utrecht University, Utrecht, Netherlands; ^2^Department of Experimental Psychology, University College London, London, United Kingdom

**Keywords:** intentional binding, sense of agency, rewards, intentional strength, intentions

## Abstract

Awareness of action is a pervasive personal experience that is crucial in understanding self-generated and other-generated actions as well as their effects. A large body of research suggests that action awareness, as measured by the magnitude of temporal binding between an action and its effect in an operant action task (i.e., intentional binding), is rooted in the human capacity to experience self-agency and establish action intentions. Whereas previous research mainly addressed the role of intentionality itself in these socially well-shared experiences, in the present study we focused specifically on one important aspect of it: the quality or strength of action intentions. We expected and established that stronger intentions increase intentional binding. Specifically, the magnitude of the binding effect, as assessed by the Libet clock task in which two actions were followed by the same neutral tone, was elevated for the action that was enacted with stronger intentions. We briefly discuss the implications of the observed role of intentional strength in temporal binding between action and effect, for promoting a better understanding and examination of how the concept of intentionality is associated with action awareness in general, and the experience of being the agent of one’s own actions in particular.

## Introduction

Intentions constitute an essential building block of human action preparation, action initiation and action awareness. Conceptually, intentions can be defined as mental representations of an individual’s upcoming volitional movement. These action representations lie at the basis of our diverse behavioral repertoire and are strongly influenced by learning. Sensorimotor processes become associated with the specific patterns of muscle activity they produce and their observable consequences on a perceptual, sensory, and motor level, allowing actions to be stored in memory together with their consequences. While these associations often operate under the radar of conscious awareness, bringing the action representation of an intention to consciousness increases the probability of preparing and initiating the associated action (Aarts et al., 2008a); as captured by the ideomotor principle (James, 1890; Greenwald, 1970; Shin et al., 2010). According to the ideomotor principle and also more recent theories such as the theory of event coding (TEC), action and effect are bound together by repetition of co-occurrence that eventually are mentally represented in terms of their causal relations (Hommel et al., 2001).

What is more, intentions do not only serve the preparation and initiation of action but also hold an important role in promoting the awareness of action and the experience of self-agency (e.g., Frith et al., 2000). Being aware of one’s actions and their consequences in the external world facilitates action planning and is crucial in sharing goals and feelings during social interaction (Baldwin and Baird, 2001; Frith, 2002). It thus does not come as a surprise that irregularities in intention formation and attribution are associated with disturbed action awareness and problems related to action planning and control (e.g., Blakemore et al., 2002; Voss et al., 2010). These irregularities are also reflected in subjective measurements of sense of agency – the feeling that one is the agent of one’s actions and their effects in the external world. Specifically, the sense of agency arises from intentional actions but is diminished for unintentional or reflexive actions (Miller and Ross, 1975; Aarts et al., 2008b; Damen et al., 2015).

Importantly, not only explicit agency reports, but also the implicit nature and manifestation of it are affected by intentionality. In an extension of the original study by Libet et al. (1983) – using an adapted version of the Wundt Clock to assess the awareness of single motor movements (e.g., lifting one’s finger) – recent studies have started to examine awareness of action in a context where movements have actual effects in the environment. They reliably find that volitional action (such as a key press) and an external stimulus (such as a tone) that follows the action at a short interval (250 ms) are shifted toward each other in temporal perception, causing the perceptual compression of the temporal interval between them. Illustrating the significance of volition, this temporal binding effect for operant actions has consistently been shown to be critically occurring for intentional (self-induced) actions and to be absent or weakened for unintentional (externally-evoked) actions, induced passively or by transcranial magnetic stimulation (TMS) (Haggard et al., 2002; Dogge et al., 2012; Borhani et al., 2017). Theoretical explanations predominantly lean on forward prediction or comparator models of motor prediction, centering around the idea of an efferent copy of the motor command being send to an internal prediction model. The incoming external sensory effect of one’s action is then being compared with the predicted sensory effect. A sense of agency is believed to arise in the case of a match between the predicted and the actual sensory effect. Similarly, in the case of a mismatch, absence of an agentic experience is expected (Blakemore et al., 2002).

Whereas being the currently most-widely accepted and plausible explanation for the evolvement of (implicit) agency experiences, these models seem to suggest an “all or nothing” mechanism, meaning that solely the absence or presence of an intention is assumed to shape and determine the manifestation of agency. Nevertheless, this construal of intentionality does not resemble reality in which individuals might hold stronger or weaker motivations to execute a specific action intention. Hence, whereas the role of intentionality of action in shaping the sense of agency has been unequivocally established, little attention has been given to the quality of these intentions. Aiming to fill this void, in the current paper we focus on this crucial aspect of intentions and investigate whether the sense of agency – as reflected by temporal binding – varies as a function of the (motivational) strength of intentions. The extent of temporal binding, we argue, does not only depend on the intention to action, but also on the strength with which people hold the intention in mind during the task or action context at hand.

In research on the philosophy of mind, intentions are often considered as entities to understand how the brain, mind, and body interact in producing observable behaviors in individuals (Searle, 1983; Dennett, 1993; Bratman, 1999). An important suggestion comes from Searle (1983), who made a distinction between prior intention (when an action is planned ahead to reach a goal or outcome) and intention in action (when an action is prepared and initiated in the task at hand) that are independently motivated. Whereas both types of intentions are important for actions to occur, intention in action is crucial for the emergence of action awareness. Following an empirical approach to predict the occurrence of intentional actions, theories in psychological science put forward the idea that intentions to engage in an action vary in strength, based on the expected value or importance of performing the action (e.g., Ajzen and Fishbein, 1977; Sheeran, 2002; Aarts et al., 2012). Actions that are expected to be of higher subjective value (such as actions that are rewarded for their execution) are thought to accommodate stronger intentions than actions that are of lower subjective value (Eitam and Higgins, 2010). Stronger intentions have been shown to be better remembered, are more readily implemented, produce more effort in the case of obstacles, and are more sustainable (Gollwitzer, 1993, 1999; Walter and Meier, 2014). In short, action intentions that carry more motivational strength are more likely to be enacted.

Recently, research has started to explore the motivational underpinnings of intentions and their role in shaping the sense of agency. Several studies have indicated that motivational manipulations such as variations in the value of action outcomes can affect temporal binding, although the direction of these effects is not always clear. Some studies found that binding increased when an individual’s intentional action caused financial gains as compared to financial losses (Takahata et al., 2012) or is followed by positive (versus negative) emotional outcomes (Yoshie and Haggard, 2013; but see: Ruys and Aarts, 2012; Moreton et al., 2017; for no differences in effects of emotional valence). Contrary though, intentional binding was shown to also increase for severely as compared to moderately negative (moral) outcomes (Moretto et al., 2011). Investigating the sense of agency in a coercion setting, Caspar et al. (2016), on the other hand, demonstrated that intentional binding was unaffected by the outcome valence when individuals intentionally inflicted financial or physical pain on another participant and were not forced to do so (Caspar et al., 2016).

Whereas suggestive, these findings do not directly speak to the role of intentional strength in action. They suggest an influence of value or importance variations of the action-outcomes on the implicit sense of agency, but they do not isolate the role of intentional strength in action from the outcome of action: participants in these studies are instructed to focus on different outcomes of actions, rather than the action itself, which confounds intention in actions with prior intentions. Put differently, the differences in binding strength could either be explained in terms of retrospective cognitive influences based on the value and type of outcomes or by an increase in strength of intention to execute the action. Hence, to test the role of strength of action intention in intentional binding, one should not vary the value and type of outcomes, but keep the outcome neutral and constant.

Circumstantial evidence for the role of intentional strength in the sense of agency was obtained by Aarts et al. (2012), who examined the effect of incidental processing of positive (vs. neutral) affect on executing action intentions in an operant action task on intentional binding. In their study, participants were briefly exposed to neutral or positive pictures before their preparation and enactment of the intention to press the spacebar on a keyboard which resulted in the presentation of one single tone. They capitalized on the notion that positive stimuli serve as a rewarding motivational drive, increasing striatal dopamine functioning. Striatal dopamine facilitates sensorimotor processes that play a role in the actual initiation of an action, the processing of outcome feedback and the experience of operant action (Dreisbach and Goschke, 2004; Aston-Jones and Cohen, 2005; Aarts et al., 2012). In line with this notion, they established that positive affect enhanced the temporal binding between the intended key press and the resulting tone. It should be noted, though, that the positive affective stimuli were irrelevant for the action intention, and hence, it remains unclear whether the indirect effect on intentional binding is the result of increased intentional strength or due to induced positive affect.

In the current research, we aimed to more directly test the influence of the motivational aspect relating to intentional strength on action awareness by addressing its influence on temporal binding. In doing so, we manipulated intentional strength directly in a task where two actions were differently rewarding, while both resulted in a neutral and fixed single outcome. Using monetary rewards to manipulate intentional strength, we induced a motivational preference for one action over another, otherwise identical, action. In comparison to other studies, we thus did not compare positive or negative action outcomes, such as gains and losses, but orthogonally manipulated the motivation of the actions itself by manipulating their expected value. In a counterbalanced within-subjects design, participants learned that a key press was more frequently rewarding (80% reward probability) than an alternative key press (20% reward probability). That is, any effects would not be due to differences in the direction of the valence of the outcome of the actions (e.g., negative or positive) but solely due to strengthened intentions to execute a specific action based on their expected rewarding quality (left versus right key press). Following, we hypothesized the more rewarding action to carry more intentional strength and thereby increasing intentional binding of the action and the neutral effect.

## Materials and Methods

### Participants and Design

Thirty-six participants^1^ (*M_age_* = 21.64, *SD_age_* = 4.42 years) with normal or corrected to normal vision took part in the experiment in exchange for monetary reimbursement. The experiment employed a 2 (target of judgment: action vs. effect) × 2 (type of trial: baseline vs. operant) × 2 (reward frequency of key: low reward frequency vs. high reward frequency) within-subjects design.

The experiment was carried out in accordance with the guidelines of the declaration of Helsinki and approved by the ethical committee of the Faculty of Social and Behavioral Sciences, Utrecht University, as part of an overarching ethical application covering a project line using the Libet clock task (ethics approval code FETC17-124). All participants gave written informed consent.

### Procedure

Participants were sat in separate cubicles, approximately 60 cm away from a computer screen. The experiment was programed using Eprime 2.0 and all instructions were provided on screen. To assure adherence to the instructions, the experimenter was present during the whole duration of the experiment.

Participants completed three different experimental stages in sequential order: a preference induction phase, a tone acquisition phase and four randomized blocks of intentional binding trials.

#### Preference Induction

The experiment utilized two different actions, pertaining to key presses (‘x’ vs. ‘n’) on a QWERTY keyboard. Key presses had different statistical probabilities to yield a monetary reward of five Eurocent. For each participant, one of the keys (‘x’ vs. ‘n’) was of ‘low reward frequency’ (20% reward probability) whereas the other was of ‘high reward frequency’ (80% reward probability), equaling average expected rewards of one and four Eurocent respectively. Participants were explicitly informed about these fixed probabilities. Key – reward probability mappings were counterbalanced across participants.

Before the beginning of the actual experiment, participants completed 40 induction trials. Each trial began with the Dutch equivalent of the statement “The following key press is worth 5 [0]) Eurocent” presented in the middle of the screen for 3000 ms, followed by a command to press either the ‘x’ (left) or ‘n’ (right) key on the keyboard. This command stayed on screen until participants had pressed the correct key. Key presses were equally distributed across the total amount of induction trials (i.e., 20 trials per key). The aim of the induction trials was to induce a distinct preference for the high reward frequency key over the low rewarding key in participants.

#### Tone Acquisition Phase

Subsequently, participants learned that both key presses have the same causal effect – a neutral 1000 Hz sinus tone of 100 ms length. To avoid confounding intentional strength with effect identity, it was emphasized that the tone (identity) would not signal reward obtainment, meaning that the reward was solely linked to the action executed.

In total, participants completed 10 practice trials (five trials per key press). Each trial began with a command to press a key which remained on screen until participants pressed the correct key. Participants’ key press was then followed by the 1000 Hz sinus tone 250 ms later. Because the purpose of these trials was only to acquaint participants with the effect of their key presses, they were not rewarded.

#### Intentional Binding Trials

Following, the actual experiment started. In total, participants completed four randomized intentional binding blocks – two baseline blocks (baseline action, baseline effect) and two operant blocks (operant action, operant effect). Each block consisted of 40 experimental trials and five practice trials which were not included in the analysis, resulting in a total of 180 trials. Trials in which participants had to press a key (baseline action, operant action, operant effect), began with a command indicating which key to press, presented in the center of the screen for 2000 ms. Both key presses were distributed equally across trials, resulting in 20 trials per key. The order of trials within a block was randomized.

Next, the clock face appeared on the screen. Trials used an adapted version of the Libet clock method (Libet et al., 1983). Participants attended to a dotted clock face, composed of 40 gray dots arranged in a circle with a diameter of six cm from the center of the screen. A black dot that moved at a period of 2560 ms per rotation served as the clock hand. The clock hand started moving from a random position on the clock face. To assure attentional focus, participants were instructed to wait with pressing the key until the clock hand had completed one full rotation and vary the moment of their key presses across trials. In operant trials, the key press was followed by the 1000 Hz sinus tone 250 ms later. In baseline action trials, the key press was not followed by the tone. In baseline effect trials, participants did not execute an action but the tone was played at a varying moment between 2560 and 5120 ms after the initial presentation of the clock face.

Before disappearing, the clock hand rotated further for 1000 ms to avoid that participants would simply report the last remembered position of the clock hand. After the disappearance of the clock hand, a prompt to judge the temporal onset of the target (i.e., key press or tone) using the mouse cursor appeared in the middle of the clock face. After a time judgment had been made, the clock face disappeared and reward feedback was presented in the middle of the screen. Reward frequencies were the same as in the preference induction phase (20 vs. 80% for the low and high reward frequency key respectively). In effect baseline trials, rewards were randomly distributed across trials, resulting in 50% of the trials being rewarded and the same averaged expected reward value as in other blocks. The inter-trial interval was set to 1000 ms.

At the end of experiment, participants filled in several questions pertaining to their demographics such as age, handedness and gender as well as their overall motivation. Finally, they were thanked for their participation and reimbursed.

## Results

### Data Set and Variables

Following Aarts et al. (2012), time judgments that exceeded or preceded the actual value of the clock hand by 640 ms (i.e., 10 dots) were excluded from analysis as they were likely to be due to inattentiveness of the participant. Less than one percent (0.31%) of the trials was excluded based on this criterium.

### Results

Separate mean judgment errors in milliseconds were calculated for the different cells of the design and subjected to a repeated measures ANOVA with target, type of trial and reward frequency of the key as within-subjects factors. Results revealed a significant main effect for target, *F*(1,35) = 4.63, *p* = 0.04, $\eta_{p}^{2}$ = 0.12. That is, the temporal perception of key presses (*M_action_* = 17.28, *SD_action_* = 49.92) was delayed whereas tones (*M_effect_* = -7.23, *SD_effect_* = 57.98) were anticipated. Moreover, the intentional binding effect was replicated, *F*(1,35) = 40.19, *p* < 0.001, $\eta_{p}^{2}$ = 0.54. Temporal perception of key presses was later in operant trials (*M_action__operant_* = 39.62, *SD_action operant_* = 70.27) than in trials in which the key press was not followed by the tone (*M_action__baseline_* = -5.06, *SD_action baseline_* = 43.78), *p* < 0.001. Similarly, tones that were initiated by a key press were perceived to occur earlier in time (*M_effect operant_* = -33.86, *SD_effect operant_* = 78.34) than tones that did not follow an action (*M_effect baseline_* = 19.46, *SD_effect baseline_* = 58.37), *p* < 0.001.

**FIGURE 1:**
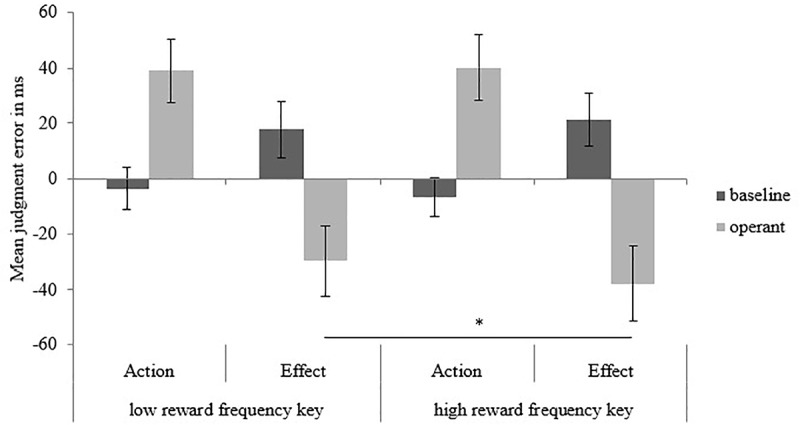
Mean judgment errors in ms for action and effect across type of trial and reward frequency of the key. Bars represent standard errors of the mean. ^∗^*p* = 0.02.

This was further qualified by a three-way interaction between target, type of trial and reward frequency of the key press, *F*(1,35) = 6.17, *p* = 0.02, $\eta_{p}^{2}$ = 0.15 (see Figure 1). Simple main effects analysis revealed that the effect was driven by tone binding. That is, the temporal perception of the action in both, baseline and operant trials, was not altered when a low or a high reward frequency key was pressed. Interestingly though, tones that were caused by an action were perceived earlier when the key was more frequently rewarding (*M_high rewarding key_* = -38.02, *SD_high rewarding key_* = 81.27) as compared to when the key was less frequently rewarding (*M_low rewarding key_* = -29.76, *SD_low rewarding key_* = 76.4), *p* = 0.02. That is, intentional binding was stronger for trials in which participants executed an action that was more frequently rewarding as compared to trials in which the action was less frequently rewarding.

## Discussion

The general importance of intentionality for the emergence of action awareness and agentic experiences has been widely established, with sense of agency being diminished when intentionality is absent. Interestingly though, little research has examined the influence of the quality of these intentions – or intentional strength – on (implicit) agency. Aiming to fill this void, in the current research we investigated how the implicit experience of self-agency changes when the operant action is enacted by stronger versus weaker intentions based on the expected value of the action. Confirming our hypothesis, temporal binding indeed increased for trials on which participants pressed a frequently rewarded key, suggesting a magnified implicit sense of agency. More specifically, the temporal onset of the outcome was significantly anticipated whereas the perception of the temporal onset of the action was not significantly altered. Thus, we were able to demonstrate that not only the presence of intentions, but also their motivational value alters the implicit processes of the sense of agency.

Our findings are in line with theoretical frameworks emphasizing the importance of motivational relevance in intention formation, such as the relevance of a representation framework (ROAR), which suggests that action representations carrying a stronger motivational value are more actively held in mind and thus also more likely to influence behavior and experiences (Eitam and Higgins, 2010). Generally, subjectively more relevant stimuli evoke brain activity significantly earlier than subjectively less relevant stimuli (e.g., Schupp et al., 2004; Schultz, 2006), which facilitates intention formation and action execution. In our experiment, the action cue for the high reward frequency key would be such a motivationally relevant stimulus, activating a strong action representation and giving rise to an enhanced efference copy. This, in return, would cause a more pronounced intentional binding effect. On a neurobiological level, this can be explained in terms of a dopaminergic boost. The action cue at the beginning of a trial carries reward-related information which causes dopaminergic activation in the ventral striatum. This dopaminergic activation facilitates the transferal of information from the pre-frontal cortex to cortical motor areas, affecting voluntary action as well as the awareness thereof (Schultz, 2006; Aarts et al., 2012).

Although speculative, such an explanation in terms of increased action preparation would also be in line with earlier research on inter-individual differences in the experience of intentionality and free will beliefs in agency contexts. Specifically, past research showed correlations between intentional awareness and brain activation related to motor preparation for individuals who are assumed to enjoy high levels of internal insight (e.g., meditators; Jo et al., 2015), as well as demonstrated that these differences can affect the implicit sense of agency. A study by Lush et al. (2016), for example, found that intentional binding was stronger for experienced meditators who find practicing mindfulness pleasurable, than for controls (Lush et al., 2016). In addition, beliefs in free will have been linked to intentional binding, which is explained in terms of a motivational or cognitive orientation toward the results of action intentions (Aarts and Van den Bos, 2011; Lynn et al., 2014). Together, these findings suggest that stronger experiences of intentions have a special status in motivating people toward preparing actions and tuning them for predicting or anticipating feedback information that will result from enacting intentions. On this view, intentional strength increases the sense of agency by motivational (action preparation) as well as cognitive (effect anticipation) processes.

There is previous research that concurs with the findings reported in the present paper. Specifically, motivation manipulations have been used to increase the value of outcomes of actions, and to test the role of such motivational enhancement on intentional binding. The general gist of these studies is that outcome importance modulates binding between action and outcome under free (voluntary) as well as coercive (involuntary) conditions of behavior (Takahata et al., 2012; Yoshie and Haggard, 2013; Caspar et al., 2016; Moreton et al., 2017). While these finding are supportive of the role of intentional strength, it is not clear whether they are due to motivational enhancement of action intentions or prior intentions. Teasing these two different accounts apart is important: they might shed light on the question whether action awareness is a direct function of the strength of intention in action and the subsequent preparation and initiation of it (Libet, 1985), or relies on a prior intention that includes information about the importance of outcomes of action. As has been argued and shown, prior intentions increase attention to, and strengthen anticipation of desired outcomes (Aarts, 2012), and readily evoke a retrospective inference process after action execution that produces post-conscious thoughts about agency and behavior (Wegner, 2003; Aarts et al., 2005). On this view, prior intentions effects on intentional binding are not the result of motivational processes *per se*, but could rather be considered as cognitive effects in which humans use causal knowledge about their action and potential effects to inform themselves about agency.

Whereas our findings favor an intentional strength account for action, we would like to address a few issues that limit the interpretation of the results of our study. The first issue relates to the absence of free choice in action selection. We wanted to ensure that intentional strength rather than preferences would modulate the intentional binding effect. Since preferences would however always confound free choice, we controlled for action preferences by instructing participants which key to press on a given trial. Nonetheless, it could be argued that this absence of free choice in action selection does not allow for, or at least negatively impacts, intention formation. Research by Barlas and Obhi (2013), for example, showed decreased intentional binding when participants had limited action alternatives (Barlas and Obhi, 2013). Nevertheless, binding, albeit decreased, did not disappear for conditions in which individuals were given moderate action selection options or no choice. Furthermore, it is important to note that the original intentional binding study (Haggard et al., 2002) established a strong and replicable intentional binding effect, even though only one key could be pressed, and no other choice alternatives that causally linked the action to the effect existed (Haggard et al., 2002). This suggests that for intentional binding to arise, it suffices to hold an intended action in mind and to choose the moment to act on this intention (Brass and Haggard, 2008). Thus, while action intentions were externally provided by instructions rather than self-generated, participants could choose to time their action that, in principle, represents the intention in action upon one’s own will (Searle, 1983).

Another related issue that needs to be addressed concerns the question of whether externally induced action intentions can be viewed as being rooted in the intentionality of action at all. To answer this question, one needs to assume that external (instructed) and internal (self-chosen) intentions differ in their effects on behavior. Whereas self-generated and externally generated intentions might differ in their phenomenological experiences, research suggests that both type of intentions can have similar effects on behavior. Research on goal-setting theory, for example, shows that self-assigned and other-assigned goals do not necessarily result in performance differences, especially when both types of goals are important (Locke and Latham, 1994, 2019). Furthermore, there is recent empirical evidence that externally induced action intentions can produce similar effects on intentional binding as self-generated action intentions (Wang et al., 2017). In an attempt to examine intentional binding effects in a Simon task, Wang et al. (2017) asked participants to respond immediately to a cue, causing a tone to occur. Thus, whereas knowledge about the causal relation between action and effect was present, both choice and timing of the action were fully pre-determined. Their findings demonstrated that even under such strong externally forced action intentions, intentional binding occurred to a similar degree as found for self-timed intention effects.

These findings raise the question of whether intentions should only be defined in terms of what, when and whether at all, or whether other factors might be more important and decisive in binding action and effect together and creating a sense of agency, such as causal beliefs and other knowledge-based predictions about action and effects (Van der Weiden et al., 2011; Dogge et al., 2012). Indeed, it has been repeatedly argued that such beliefs and knowledge play a pivotal role in the way people form intentions and consider themselves as active agents that can engage in action performance to realize their intentions and goals (e.g., Bandura, 1977; Ajzen, 1991). Thus, whereas rewards might create stronger intentions for future actions during learning, the enhanced intentional strength itself might render causal beliefs more salient, supporting people to more strongly predict the effects of their actions, leading to a stronger sense of agency.

## Conclusion

We observed that actions that are more rewarding led to a stronger sense of agency in an intentional binding task. These effects could not be attributed to preferences for actions or their effects. The mere fact that stronger action intentions led to stronger intentional binding suggests that intentional strength is associated with action awareness: individuals might become more readily aware of actions that are furnished with strong intentions. We do not know yet whether this intention-action-awareness relationship results from motivational or cognitive mechanisms. However, we hope and believe that the concept of intentional strength might offer an important addition to the study of intentional binding in general, and more specifically to the understanding of how intentionality is represented in the conscious awareness that people have in experiencing themselves as the agent of their own behavior.

## Ethics Statement

This study was carried out in accordance with the Declaration of Helsinki. All subjects gave written informed consent. The study was approved by the faculty ethics review board (ethics approval code: FETC17-124).

## Author Contributions

SA, RC, and HA conceived the idea and planned the experiment and wrote the manuscript. SA and RC programed the experiment. SA collected the data and analyzed and interpreted the data.

## Conflict of Interest Statement

The authors declare that the research was conducted in the absence of any commercial or financial relationships that could be construed as a potential conflict of interest.

## Footnotes

1 The sample size was therefore based on similar study in our lab, *n* = 28 (Aarts et al., 2012). Since most previous studies testing moderator effects on intentional binding used smaller samples, we aimed to ascertain that we test a sufficiently large sample.

## References

[B1] Aarts H. (2012). “Goals, motivated social cognition and behavior,” in *The SAGE Handbook of Social Cognition*, eds Fiske S. T. Neil Macrae C. (London: SAGE), 75–79.

[B2] Aarts H. Bijleveld E. Custers R. Dogge M. Deelder M. Schutter D. (2012). Positive priming and intentional binding: eye-blink rate predicts reward information effects on the sense of agency. *Soc. Neurosci.* 7 105–112. 10.1080/17470919.2011.590602 21936738

[B3] Aarts H. Custers R. Marien H. (2008a). Preparing and motivating behavior outside of awareness. *Science* 319 1639–1639. 10.1126/science.1150432 18356517

[B4] Aarts H. Dijksterhuis A. Dik G. (2008b). “Goal contagion: inferring goals from others’ actions - and what it leads to,” in *Handbook of Motivation Science*, eds Shah J. Y. Gardner W. (New York, NY: Guilford).

[B5] Aarts H. Custers R. Wegner D. M. (2005). On the inference of personal authorship: enhancing experienced agency by priming effect information. *Conscious. Cogn.* 14 439–458. 10.1016/j.concog.2004.11.001 16091264

[B6] Aarts H. Van den Bos K. (2011). On the foundations of beliefs in free will: intentional binding and unconscious priming in self-agency. *Psychol. Sci.* 22 532–537. 10.1177/0956797611399294 21317370

[B7] Ajzen I. (1991). The theory of planned behavior. *Organ. Behav. Hum. Decis. Process.* 50 179–211.

[B8] Ajzen I. Fishbein M. (1977). Attitude-behavior relations: a theoretical analysis and review of empirical research. *Psychol. Bull.* 84 888–918. 10.1037//0033-2909.84.5.888

[B9] Aston-Jones G. Cohen J. D. (2005). An integrative theory of locus coeruleus-norepinephrine function: adaptive gain and optimal performance. *Annu. Rev. Neurosci.* 28 403–450. 10.1146/annurev.neuro.28.061604.135709 16022602

[B10] Baldwin D. A. Baird J. A. (2001). Discerning intentions in dynamic human action. *Trends Cogn. Sci.* 5 171–178. 10.1016/s1364-6613(00)01615-6 11287271

[B11] Bandura A. (1977). Self-efficacy: toward a unifying theory of behavioral change. *Psychol. Rev.* 84 191–215. 10.1037/0033-295x.84.2.191 847061

[B12] Barlas Z. Obhi S. (2013). Freedom, choice, and the sense of agency. *Front. Hum. Neurosci.* 7:514. 10.3389/fnhum.2013.00514 24009575 PMC3756740

[B13] Blakemore S. J. Wolpert D. M. Frith C. D. (2002). Abnormalities in the awareness of action. *Trends Cogn. Sci.* 6 237–242. 10.1016/s1364-6613(02)01907-1 12039604

[B14] Borhani K. Beck B. Haggard P. (2017). Choosing, doing, and controlling: implicit sense of agency over somatosensory events. *Psychol. Sci.* 28 882–893. 10.1177/0956797617697693 28488908

[B15] Brass M. Haggard P. (2008). The what, when, whether model of intentional action. *Neuroscientist* 14 319–325. 10.1177/1073858408317417 18660462

[B16] Bratman M. E. (1999). *Faces of Intention: Selected Essays on Intention and Agency.* Cambridge: Cambridge University Press.

[B17] Caspar E. A. Christensen J. F. Cleeremans A. Haggard P. (2016). Coercion changes the sense of agency in the human brain. *Curr. Biol.* 26 585–592. 10.1016/j.cub.2015.12.067 26898470 PMC4791480

[B18] Damen T. G. Van Baaren R. B. Brass M. Aarts H. Dijksterhuis A. (2015). Put your plan into action: the influence of action plans on agency and responsibility. *J. Pers. Soc. Psychol.* 108 850–866. 10.1037/pspa0000024 25984787

[B19] Dennett D. C. (1993). *Consciousness Explained.* London: Penguin.

[B20] Dogge M. Schaap M. Custers R. Wegner D. M. Aarts H. (2012). When moving without volition: implied self-causation enhances binding strength between involuntary actions and effects. *Conscious. Cogn.* 21 501–506. 10.1016/j.concog.2011.10.014 22115726

[B21] Dreisbach G. Goschke T. (2004). How positive affect modulates cognitive control: reduced perseveration at the cost of increased distractibility. *J. Exp. Psychol. Learn. Mem. Cogn.* 30 343–353. 10.1037/0278-7393.30.2.343 14979809

[B22] Eitam B. Higgins E. T. (2010). Motivation in mental accessibility: relevance of a representation (ROAR) as a new framework. *Soc. Pers. Psychol. Compass* 4 951–967. 10.1111/j.1751-9004.2010.00309.x 21116462 PMC2992320

[B23] Frith C. (2002). Attention to action and awareness of other minds. *Conscious. Cogn.* 11 481–487. 10.1016/s1053-8100(02)00022-3 12470618

[B24] Frith C. D. Blakemore S. J. Wolpert D. M. (2000). Abnormalities in the awareness and control of action. *Philos. Trans. R. Soc. Lond. Ser. B Biol. Sci.* 355 1771–1788. 10.1098/rstb.2000.0734 11205340 PMC1692910

[B25] Gollwitzer P. M. (1993). Goal achievement: the role of intentions. *Eur. Rev. Soc. Psychol.* 4 141–185. 10.1080/14792779343000059

[B26] Gollwitzer P. M. (1999). Implementation intentions: strong effects of simple plans. *Am. Psychol.* 54 493–503. 10.1037//0003-066x.54.7.493

[B27] Greenwald A. G. (1970). Sensory feedback mechanisms in performance control: with special reference to the ideo-motor mechanism. *Psychol. Rev.* 77 73–90. 10.1037/h0028689 5454129

[B28] Haggard P. Clark S. Kalogeras J. (2002). Voluntary action and conscious awareness. *Nat. Neurosci.* 5 382–385. 10.1038/nn827 11896397

[B29] Hommel B. Müsseler J. Aschersleben G. Prinz W. (2001). The theory of event coding (TEC): a framework for perception and action planning. *Behav. Brain Sci.* 24 849–878. 10.1017/s0140525x01000103 12239891

[B30] James W. (1890). Attention. *princ. psychol.* 1 402–458. 10.1037/10538-011

[B31] Jo H. G. Hinterberger T. Wittmann M. Schmidt S. (2015). Do meditators have higher awareness of their intentions to act? *Cortex* 65 149–158. 10.1016/j.cortex.2014.12.015 25706808

[B32] Libet B. (1985). Unconscious cerebral initiative and the role of conscious will in voluntary action. *Behav. Brain Sci.* 8 529–539. 10.1017/S0140525X00044903

[B33] Libet B. Gleason C. A. Wright E. W. Pearl D. K. (1983). “Time of conscious intention to act in relation to onset of cerebral activity (readiness-potential),” in *Neurophysiology of Consciousness*, (Boston, MA: Birkhäuser),249–268. 10.1007/978-1-4612-0355-1_15 6640273

[B34] Locke E. Latham G. (1994). “Goal-setting theory. Organizational behavior 1” in *Essential Theories of Motivation and Leadership*, ed. Miner J. B. (New York, NY: Routledge), 159–183.

[B35] Locke E. A. Latham G. P. (2019). The development of goal setting theory: a half century retrospective. *Motiv. Sci.* 10.1037/mot0000127

[B36] Lush P. Parkinson J. Dienes Z. (2016). Illusory temporal binding in meditators. *Mindfulness* 7 1416–1422. 10.1007/s12671-016-0583-z 27909466 PMC5107189

[B37] Lynn M. T. Muhle-Karbe P. S. Aarts H. Brass M. (2014). Priming determinist beliefs diminishes implicit (but not explicit) components of self-agency. *Front. Psychol.* 5:1483. 10.3389/fpsyg.2014.01483 25566155 PMC4268906

[B38] Miller D. T. Ross M. (1975). Self-serving biases in the attribution of causality: fact or fiction? *Psychol. Bull.* 82 213–225. 10.1037/h0076486

[B39] Moreton J. Callan M. J. Hughes G. (2017). How much does emotional valence of action outcomes affect temporal binding? *Conscious. Cogn.* 49 25–34. 10.1016/j.concog.2016.12.008 28107726

[B40] Moretto G. Walsh E. Haggard P. (2011). Experience of agency and sense of responsibility. *Conscious. Cogn.* 20 1847–1854. 10.1016/j.concog.2011.08.014 21920776

[B41] Ruys K. I. Aarts H. (2012). I didn’t mean to hurt you! unconscious origins of experienced self-agency over others’ emotions. *Emotion* 12 132–141. 10.1037/a0023161 21707153

[B42] Schultz W. (2006). Behavioral theories and the neurophysiology of reward. *Annu. Rev. Psychol.* 57 87–115. 10.1146/annurev.psych.56.091103.070229 16318590

[B43] Schupp H. T. Öhman A. Junghöfer M. Weike A. I. Stockburger J. Hamm A. O. (2004). The facilitated processing of threatening faces: an ERP analysis. *Emotion* 4 189–200. 10.1037/1528-3542.4.2.189 15222855

[B44] Searle J. R. (1983). *Intentionality: An Essay in the Philosophy of Mind.* Cambridge: Cambridge university press.

[B45] Sheeran P. (2002). Intention—behavior relations: a conceptual and empirical review. *Eur. Rev. Soc. Psychol.* 12 1–36. 10.1002/0470013478.ch1

[B46] Shin Y. K. Proctor R. W. Capaldi E. J. (2010). A review of contemporary ideomotor theory. *Psychol. Bull.* 136 943–974. 10.1037/a0020541 20822210

[B47] Takahata K. Takahashi H. Maeda T. Umeda S. Suhara T. Mimura M. (2012). It’s not my fault: postdictive modulation of intentional binding by monetary gains and losses. *PLoS One* 7:e53421. 10.1371/journal.pone.0053421 23285293 PMC3532346

[B48] Van der Weiden A. Aarts H. Ruys K. I. (2011). Prime and probability: causal knowledge affects inferential and predictive effects on self-agency experiences. *Conscious. Cogn.* 20 1865–1871. 10.1016/j.concog.2011.09.007 21963403

[B49] Voss M. Moore J. Hauser M. Gallinat J. Heinz A. Haggard P. (2010). Altered awareness of action in schizophrenia: a specific deficit in predicting action consequences. *Brain* 133 3104–3112. 10.1093/brain/awq152 20685805

[B50] Walter S. Meier B. (2014). How important is importance for prospective memory? A review. *Front. Psychol.* 5:657. 10.3389/fpsyg.2014.00657 25018743 PMC4071817

[B51] Wang Y. Damen T. G. Aarts H. (2017). Uncovering effects of self-control and stimulus-driven action selection on the sense of agency. *Conscious. Cogn.* 55 245–253. 10.1016/j.concog.2017.09.005 28942359

[B52] Wegner D. M. (2003). The mind’s best trick: how we experience conscious will. *Trends Cogn. Sci.* 7 65–69. 10.1016/s1364-6613(03)00002-0 12584024

[B53] Yoshie M. Haggard P. (2013). Negative emotional outcomes attenuate sense of agency over voluntary actions. *Curr. Biol.* 23 2028–2032. 10.1016/j.cub.2013.08.034 24094850

